# The time-course of EEG alpha power changes in creative ideation

**DOI:** 10.3389/fnhum.2014.00310

**Published:** 2014-05-13

**Authors:** Daniela Schwab, Mathias Benedek, Ilona Papousek, Elisabeth M. Weiss, Andreas Fink

**Affiliations:** Institute of Psychology, University of GrazGraz, Austria

**Keywords:** EEG, alpha power, creative ideation, divergent thinking, time-course

## Abstract

Increases in EEG alpha power during creative ideation are among the most consistent findings in the neuroscientific study of creativity, but existing studies did not focus on time-related changes of EEG alpha activity patterns during the process of creative ideation so far. Since several cognitive processes are involved in the generation of creative ideas, different EEG correlates may result as a function of time. In this study we addressed this crucial point. Forty-five participants worked on the “Alternative Uses Task” while the EEG was recorded and changes in task-related power (relative to rest) in the upper-frequency band (10–12 Hz) for three isochronous time intervals of the idea generation period were determined. Alpha power changes during idea generation followed a characteristic time course: we found a general increase of alpha power at the beginning of idea generation that was followed by a decrease and finally by a re-increase of alpha prior to responding that was most pronounced at parietal and temporal sites of the right hemisphere. Additionally, the production of more original ideas was accompanied by increasing hemispheric asymmetry (more alpha in the right than left hemisphere) with increasing duration of the idea generation period. The observed time course of brain activity may reflect the progression of different but well-known stages in the idea generation process: that is the initial retrieval of common and old ideas followed by the actual generation of novel and more creative ideas by overcoming typical responses through processes of mental simulation and imagination.

Research on creativity—commonly defined as “the ability to produce work that is both novel and appropriate” (Sternberg and Lubart, 1996, p. 677)—has recently attracted great attention in psychology and particularly in the field of neuroscience. It is often dated back to Guilford’s well-known presidential address at the American Psychological Association, where he highlighted the crucial importance of research on creativity which has been neglected for a comparatively long period of time (Guilford, 1950). Since then, creativity as a research topic has become more and more popular and has been addressed in various scientific disciplines by adopting a broad variety of different perspectives and methodological approaches (cf. Simonton, 2000; Runco, 2004).

The underlying neural basis of creative cognition is a major area of interest in creativity research. Based on the conceptualization of creative thought as the combination or interplay of different cognitive processes (e.g., Guilford, 1950), it is unlikely that there is just one “center of creativity” in the brain (cf. Dietrich, 2004). To come up with a creative idea, people need to be aware of a certain problem, to be able to analyze the situation and to redefine it. Then, possible alternatives or solutions resulting from cognitive processes such as flexible thinking have to be developed and are finally needed to be evaluated (cf. Guilford, 1950). Moreover, the generation of a novel idea is thought to involve the meaningful recombination of previously unrelated semantic concepts or frames of thought (Mednick, 1962; Koestler, 1964; Benedek et al., 2012). Thus, various cognitive processes such as attention, memory retrieval, working memory etc. could be considered as being crucially involved in the idea generation process. In this particular context it is hypothesized that the same combination of neural networks that is recruited in non-creative cognition (e.g., working memory) is implicated in creative cognition as well (e.g., Cabeza and Nyberg, 2000; Dietrich, 2004).

As a consequence of the broad variety of different approaches and methods that were used to investigate creativity, previous findings on neural correlates of creativity have been heterogeneous and often inconsistent (cf. Arden et al., 2010; Dietrich and Kanso, 2010). In reviewing relevant findings in this field, Dietrich and Kanso (2010) concluded that the results differ widely because of the diversity of possible assessments of creativity, including varying research foci (divergent thinking, artistic creativity, problem solving with insight) and neuroscientific methods (e.g., EEG, fMRI). Fink and Benedek (2012) suggested that comparing findings across different studies might be more reasonable by specifically looking at studies in which similar tasks and measurement methods were used. In doing so, Fink and Benedek (2012) reviewed EEG studies which specifically focused on the relationship between creative ideation (i.e., divergent thinking) and power in the alpha frequency band (8–12 Hz). Their review revealed robust evidence of EEG alpha power being particularly sensitive to various creativity-related demands: alpha power varies as a function of creativity-related task demands (the more creative a task the higher the level of alpha), as a function of originality (higher originality is accompanied by more alpha), and as a function of an individuals’ creativity level (more alpha in higher creative individuals, see Fink and Benedek, 2012, 2013). Additionally, alpha power has also been observed to increase as a result of verbal creativity interventions (Fink et al., 2006, 2011). On the basis of these findings Fink and Benedek (2012) concluded that the observed alpha findings are among the most consistent findings in the neuroscientific study of creativity, and we might therefore assume that the study of alpha power changes is a valuable and powerful tool to study brain activity patterns during the process of creative idea generation.

Traditionally, increases in EEG alpha power (hereinafter referred to as alpha synchronization) have been interpreted as sign of “cortical idling” (cf., Pfurtscheller et al., 1996). More recent studies suggested that alpha synchronization may indicate a state of high internal processing demands that are characterized by “the absence of bottom-up processing (Ray and Cole, 1985; Cooper et al., 2003) and thus can be classified a pure form of top-down activity” (von Stein and Sarnthein, 2000, p.311). This top-down process may have an attentional control function leading to inhibition of task-irrelevant stimuli (Klimesch et al., 2007). Therefore, alpha synchronization is probably associated with selective and active cognitive processing (Sauseng et al., 2005).

Alpha synchronization observed during creative ideation, which has consistently been observed over the prefrontal and temporo-parietal cortex (Fink and Benedek, 2012), may also reflect active cognitive processing and focused internal attention (Fink et al., 2009a; Benedek et al., 2011; Fink and Benedek, 2012; Jauk et al., 2013). Finding a creative solution requires effective memory retrieval and effective working memory processing for knowledge modulation (activation, inhibition and combination of knowledge; see e.g., Heilman et al., 2003; Dietrich, 2004). In addition, alpha synchronization at parietal sites of the right hemisphere seems to play an important role in creating ideas with high originality. In previous studies, stronger alpha synchronization at right parietal sites was found in higher creative individuals compared to less creative individuals (e.g., Martindale and Hines, 1975; Jaušovec, 2000; Razumnikova, 2007; Fink et al., 2009a,b).

Besides neuroscientific research on creativity, theoretical models were postulated to describe creative cognition and its possible stages or phases. The number of phases that are assumed as being implicated in the creative process vary (e.g., Wallas, 1926; Finke et al., 1992; Csikszentmihalyi, 1996). Exemplarily, in the “Geneplore” model two phases are hypothesized (Finke et al., 1992). According to this model, creative cognition is considered as the result of processing in circuits that consist of (reoccurring) generative and exploratory phases (Finke et al., 1992). The authors assume that idea generation starts with the construction of mental representations (generative phase) followed by interpretation and modification processes (exploratory phase). Other models propose four (preparation, incubation, illumination and verification; Wallas, 1926) or even five stages (preparation, incubation, insight, evaluation and elaboration; Csikszentmihalyi, 1996).

There were also attempts to examine the different strategies and cognitive processes involved in divergent thinking (i.e., creative idea generation) tasks. Gilhooly et al. (2007) performed a verbal protocol analysis of the alternate uses task (i.e., a common divergent thinking task that requires to generate creative new uses for common objects) and categorized the processes reported during the task. The study revealed that initial ideas were largely based on the retrieval of known uses from memory, whereas later ideas were based on more complex strategies such as focusing on specific object properties or using imagery (cf. Benedek et al., 2014a). As a result, ideas are generally observed to become more creative over time (e.g., Beaty and Silvia, 2012; Benedek and Neubauer, 2013). It was proposed that the generation of creative ideas involves executive processes such as prepotent response inhibition in order to overcome initial dominant responses and to provide top-down control of attention during strategic semantic search processes (Gilhooly et al., 2007; Beaty and Silvia, 2012; Benedek et al., 2012). This notion is further substantiated by studies reporting a close link of divergent thinking ability with higher-order cognitive ability (Nusbaum and Silvia, 2011; Beaty and Silvia, 2013; Jauk et al., 2013, 2014).

Until now, there is only very little evidence on the time-course of creativity-related processes on the neurophysiological level. Studies which investigated neural correlates of insightful problem solving (i.e., problem solving accompanied by subjective experience of insight or the “AHA moment”) reported an increase of alpha power at parietal and temporal sites of the right hemisphere shortly before solving tasks with insight (Jung-Beeman et al., 2004; Kounios et al., 2006; Sandkühler and Bhattacharya, 2008; Sheth et al., 2009). Time-related neural responses during the process of creative ideation (i.e., the generation of creative ideas) are yet unknown.

The aim of this study was therefore to investigate the time-course of EEG alpha power during the process of creative ideation. Theoretical accounts assume the involvement of different phases or processes in creative idea generation (e.g., Wallas, 1926; Finke et al., 1992; Csikszentmihalyi, 1996), which is why we expect different patterns of EEG alpha power as a function of time. In order to address this question we reanalyzed a recent EEG study of our laboratory in which alpha power activity during idea generation was investigated (Fink et al., 2011). In that study participants were required to generate alternative uses to everyday objects (i.e., AU task) after being stimulated via affective and cognitive interventions, whereas during the control condition they performed the task without any intervention. As a measure of brain activity, only alpha power during the entire idea generation period was quantified. In the present study we focused on the time course of alpha power during different phases of idea generation. Therefore we determined alpha power estimates for three subsequent time intervals within the idea generation period of the AU task in order to get first insights whether or to which extent alpha power during creative ideation changes as a function of time. Based on recent evidence on the relationship between alpha power and creative ideation (Fink and Benedek, 2012), we expected time-related alterations of alpha power at prefrontal as well as over parietal and temporal sites (of the right hemisphere) as a possible indication of varying demands on memory retrieval and working memory processing during the process of idea generation. In addition, we also investigated whether ideas of varying originality show different time-related changes of EEG alpha power. Recent research revealed that alpha power at right parietal sites may be important in originality (Fink and Benedek, 2012), and given the manifold cognitive processes that are required to generate originality, we expected that the association between alpha power and originality may also vary as a function of time.

## Method

### Participants

Forty-eight students participated in the study (cf. Fink et al., 2011). Three participants had to be excluded from data analyses due to technical problems during EEG recording. The final sample included 45 participants (22 women, 23 men) aged 18 to 32 years (*M* = 23.09, *SD* = 3.48). They were right-handed as assessed by a standardized handedness test (Steingrüber and Lienert, 1971; Papousek and Schulter, 1999). The participants indicated no history of medical, psychiatric or neurological disorders or treatment that could have interfered with any of the behavioral and neurophysiological measures. This study was approved by the local ethics committee of the University of Graz.

### Task and procedure

Participants worked on the Alternative Uses (AU) task (cf. Wilson et al., 1953; Fink et al., 2007) while the EEG was measured. In the AU conventional everyday objects such as “shoes” or “toothpaste” were presented on the screen, and participants were instructed to come up with original and unconventional uses for these objects. Participants worked on three different experimental conditions, each of them consisting of 15 items. In two experimental conditions participants were stimulated via brief cognitive and affective intervention during idea generation (see Fink et al., 2011) while in the control condition no intervention was applied. The data presented in this paper are based on the 15 AU items of the control condition. At the beginning, two 2 min EEG sequences under resting conditions were recorded, the first with eyes closed, the second with eyes open. Before EEG recording, participants were carefully instructed how to perform the AU task.

**Figure 1:**
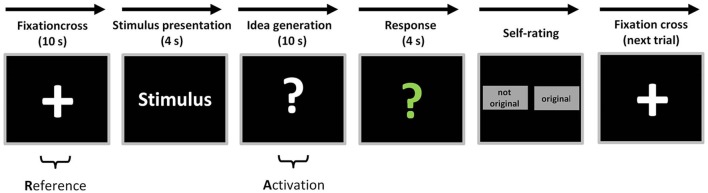
**Overview of experimental task and measurement intervals**. Each AU trial started with a fixation cross for the duration of 10 s (reference period) before the stimulus word appeared for 4 s on the screen. During the presentation of the white question mark participants had to think about useful and original ideas for the stimulus for a time period of 10 s (activation period). Subsequently, the question mark changed its color into green and the participants had to articulate their most original idea within 4 s. Finally, participants were asked to evaluate their response either as “original” or “not original” via mouse click on the corresponding choice box on the screen (figure adapted from Fink et al., 2011).

The AU task started with the presentation of a fixation cross for the duration of 10 s (reference period, see Figure 1). Then the stimulus word (everyday object) appeared for 4 s on the screen. Subsequently, a white question mark appeared on the screen, indicating that participants had to think about useful and original ideas for the given stimulus for a time period of 10 s (idea generation period). Afterwards, the question mark changed its color into green signalizing the participants to articulate their idea within a time period of 4 s. For further analysis, the oral responses were recorded and transcribed (cf., Fink et al., 2007). At the end of each trial, participants were asked to evaluate their response either as “original” or “not original” via mouse click on the corresponding choice box on the screen. Then the next trial started. The presentation of the AU stimuli during EEG recording was fully randomized.

### Analysis of AU performance data

Originality of creative idea generation in the AU task was assessed via self-rating and external ratings (Fink et al., 2007; Benedek et al., 2013). For the external ratings, nine experienced raters were instructed to judge the originality of each response of a participant on a five-point rating scale ranging from 1 (“highly original”) to 5 (“not original at all”). Inter-rater agreement was satisfactory (*intra-class correlation coefficient* = 0.76). The ratings for each response were averaged over raters to obtain a measure of idea originality. For each participant, we then divided (via median-split) the total number of ideas into trials in which more vs. less original ideas (assessed via external ratings) were generated. This distinction was used as a within-subjects factor in further analysis. A paired samples *t*-test (*t*_44_ = 24.28; *p* < 0.001; *M*_less original ideas_ = 3.54, *M*_more original ideas_ = 2.66) revealed that the average score of more original ideas was significantly lower (denoting higher originality) than the average score of less original ideas.

### EEG data recording and analysis

The EEG was measured with a BrainVision BrainAmp Research Amplifier (Brain Products) with Ag/AgCl electrodes and a stretchable electrode cap from the following 19 positions after the international 10–20 system (Jasper, 1958): FP_1_, FP_2_, F_3_, F_7_, F_*Z*_, F_4_, F_8_, T_7_, C_3_, C_*Z*_, C_4_, T_8_, P_7_, P_3_, P_*Z*_, P_4_, P_8_, O_1_, O_2_. The midline electrodes (F_*Z*_, C_*Z*_, P_*Z*_) were not included in the statistical analysis (given that we were also interested in potential hemispheric differences). The ground electrode was located at FP_*Z*_, the reference electrode was placed on the nose. To register eye movements, an electrooculogram (EOG) was recorded bipolarly between two electrodes diagonally placed above and below the inner and the outer canthus of the right eye. The EEG signals were filtered between 0.1 Hz and 100 Hz. An additional 50 Hz notch filter was applied. Electrode impedances were kept below 5 kΩ for the EEG and below 10 kΩ for the EOG. All signals were sampled at a frequency of 500 Hz.

EEG data were preprocessed by removing drifts and low pass filtering (50 Hz). The data were visually inspected for artifacts and artifactual epochs caused by muscle tension, eye blinks or eye movements were excluded from further analyses. Also, only trials with a valid answer were included in statistical analysis. In a next step, EEG signals were filtered by applying an FFT filter for the upper alpha frequency band (10 and 12 Hz). Power estimates were obtained by squaring filtered EEG signals, and then band power values (μV^2^) were (horizontally) averaged for each single trial.

As in previous studies (e.g., Fink et al., 2009a, 2011), we quantified task-related power (TRP) changes in the upper alpha band during creative ideation. In computing TRP, 8 s time segments (out of 10 s) in the middle of the reference period (starting 1 s after the onset of the fixation cross) as well as the 8 s segment in the middle of the activation period (starting 1 s after the onset of the white question mark, cf. Figure 1) were used. The TRP for each electrode position (i) was computed according to the formula: TRP(logPow_*i*_) = log [Pow_*i* activation_] − log [Pow_*i* reference_] (Pfurtscheller, 1999). That means that the (log-transformed) power during the reference period (fixation cross) was subtracted from the (log-transformed) power during the activation period (creative ideation). Hence, increases in power from the reference to the activation period are reflected in positive values (i.e., referred to as alpha synchronization) whereas negative values indicate decreases in power (i.e., desynchronization). To investigate the time-course of TRP the 8-s idea generation intervals were splitted into three isochronous time intervals of 2.67 s each.

TRP values were analyzed using repeated measurement ANOVA in considering the factors ORIGINALITY (more vs. less original ideas), TIME (interval 1 [1–3.67 s], interval 2 [3.67–6.33 s], interval 3 [6.33–9 s]), HEMISPHERE (left vs. right), and POSITION (eight positions in each hemisphere) as within-subjects variables. *Post-hoc* pairwise comparisons were performed using Tukey’s HSD. In case of violations of sphericity assumptions, the multivariate approach to the repeated measurements variables was used (Vasey and Thayer, 1987) and Bonferroni *post-hoc* tests (for ε < 0.70) were used.

## Results

The ANOVA yielded significant main effects of TIME *F*_2,88_ = 6.81, *p* < 0.00, $\eta_{p}^{2}=0.13$, HEMISPHERE (*F*_1,44_ = 24.68, *p* < 0.00, $\eta_{p}^{2}=0.36$) and POSITION (*F*_7,38_ = 2.90, *p* < 0.01, $\eta_{p}^{2}=0.11$) as well as significant interaction effects of TIME * HEMISPHERE (*F*_2,88_ =17.70, *p* <0.00, $\eta_{p}^{2}=0.29$, TIME * POSITION (*F*_14,31_ = 2.48, *p* < 0.02, $\eta_{p}^{2}=0.08$), HEMISPHERE * POSITION (*F*_7,38_ = 4.95, *p* < 0.00, $\eta_{p}^{2}=0.13$) and TIME * HEMISPHERE * POSITION (*F*_14,31_ = 2.63, *p* < 0.01, $\eta_{p}^{2}=0.07$).

**Figure 2:**
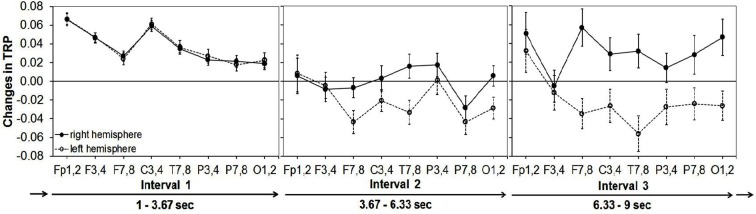
**Means and standard error bars of task-related alpha power changes (10–12 Hz) during creative idea generation for eight cortical areas of the right vs. the left hemisphere** (abbreviations for cortical sites after the international 10–20 system (Jasper, 1958): FP = frontopolar, F = frontal, C = central, P = parietal, O = occipital, T = temporal; odd numbers stand for cortical sites of the left hemisphere, even numbers for corresponding cortical sites of the right hemisphere).

**Figure 3:**
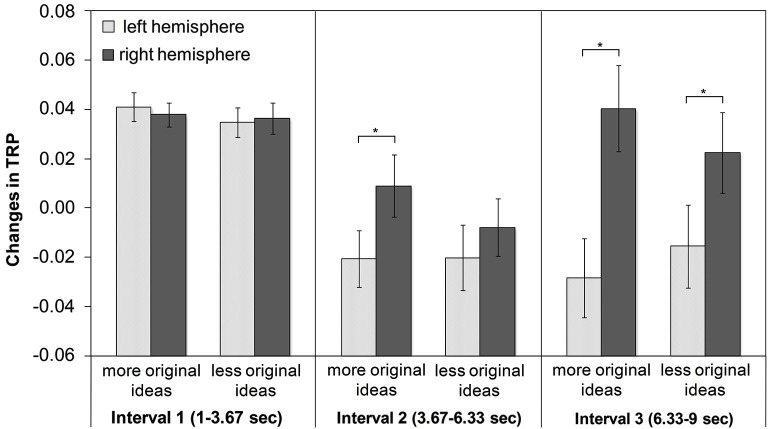
Means and standard error bars of task-related alpha power changes (10–12 Hz) during creative idea generation for more vs. less original ideas (* Tukey HSD: ***p* < 0.01)**

Alpha synchronization was stronger in the right than in the left hemisphere, a finding that was most pronounced at frontocentral and posterior cortical sites. Regarding the time-course of creative ideation, a characteristic trend of TRP was observed: as depicted in Figure 2, comparatively strong alpha synchronization occurred in the first time interval of idea generation. Then, alpha power decreased (and mostly desynchronized) during the middle time segment of the idea generation period, especially over left frontal, temporal and parietal sites. In the last time interval, alpha synchronization increased again, particularly at right-hemispheric sites (see Figure 2). With respect to idea originality, two significant interaction effects emerged: ORIGINALITY * HEMISPHERE (*F*_1,44_ = 4.35, *p* < 0.05, $\eta_{p}^{2}=3.10$) and ORIGINALITY * TIME * HEMISPHERE (*F*_2,88_ = 3.10, *p* < 0.05, $\eta_{p}^{2}=0.07$). The generally stronger increase in TRP in the right hemisphere was more pronounced for trials with more (vs. less) original ideas. The three-way interaction between originality, time and hemisphere is shown in Figure 3. During the first time interval both more and less original ideas were associated with comparatively strong alpha synchronization, similarly for both hemispheres. In the second time interval, more original ideas were associated with hemispheric asymmetry in alpha synchronization, with more TRP at right-than left-hemispheric sites. Interestingly, this asymmetry even increased at the third time interval of the idea generation period.

## Discussion

The findings of this study suggest that the process of creative ideation is characterized by a distinctive pattern of task-related EEG alpha power changes as a function of time. Specifically, the findings revealed a rather strong increase of alpha power at the beginning of idea generation, followed by a decrease in the middle time interval and a final re-increase of alpha that was confined to the right hemisphere. The time-course of creative ideation was also accompanied by a clear-cut pattern of increasing hemispheric lateralization: at left-hemispheric sites, alpha TRP showed a steady decline over time, whereas in the right hemisphere alpha TRP rather seemed to follow a U-shaped function. Moreover, we found significant effects related to the originality of ideas: while at the beginning of idea generation virtually no differences between more vs. less original ideas were found, at later time intervals more original ideas were associated with increases in right-, relative to left-hemispheric alpha power.

The idea generation task was preceded by a period of stimulus presentation in which the given stimulus (i.e., AU item) was read and encoded. After that, the actual idea generation took place. The initial phase of creative idea generation is typically characterized by the retrieval of dominant responses from memory such as common uses for the presented objects (Gilhooly et al., 2007; Benedek et al., 2014a). Alpha band activity is thought to be related to the controlled access and retrieval from memory (Klimesch et al., 2007; Klimesch, 2012), and this process has not been associated with hemispheric lateralization of alpha power, or, if any, with a tendency for a left-over-right difference (Klimesch et al., 1999). It appears thus reasonable to assume that the initial pattern of bilateral alpha synchronization indicates the typical first phase during idea generation, namely the recall of common ideas from memory.

After this initial process of creative idea generation, a general relative decrease of alpha power was observed and also a beginning hemispheric lateralization, as this decrease was less pronounced at right-hemispheric cortical sites. This lateralization then further increased in the last time-interval, driven by a re-increase of right-hemispheric TRP that was most pronounced at frontal and posterior cortical sites. Alpha synchronization over the right posterior cortex has been observed as being specific for creative thought (Fink et al., 2009a,b; Benedek et al., 2011; Jauk et al., 2012) and has been interpreted as a sign of focused internally-directed attention in order to facilitate imaginative processes during creative thought (Benedek et al., 2011; Fink and Benedek, 2012, 2013). In the context of our findings, the middle time-interval may represent a stage of the creative process during which basic retrieval from memory is increasingly receding (as indicated by reduced bilateral alpha synchronization), at the same time paving the way for more creative, imaginative thought processes. The latter would require directed and specific memory search in semantic networks, probably mediated by alpha desynchronization over left frontal, temporal and parietal sites, accompanied by a diffuse pattern of alpha synchronization over right-hemispheric regions. For example, Jensen et al. (2002) reported a strong alpha synchronization at posterior sites of the right-hemisphere especially at the end of working on a working memory task and stated “*that the tight temporal regulation of alpha provide strong evidence that the alpha generation system is directly or indirectly linked to the circuits responsible for working memory (p.877)”*. As creative idea generation proceeds, more complex processes such as mental simulation and the generation of mental images (of new and more creative object uses) are presumably going to take place (Gilhooly et al., 2007). These processes may be especially sensitive to interference from distracting irrelevant external stimulation and thus be accompanied by an even stronger right-hemispheric EEG alpha synchronization reflecting a process of task shielding (Jensen et al., 2002; Benedek et al., 2014b).

Most strikingly, the described pattern of results was more pronounced in higher original ideas compared to less original ideas (see Figure 3). A stronger alpha synchronization at right-hemispheric sites for higher (vs. less) original individuals or ideas has been previously reported (e.g., Grabner et al., 2007; Fink et al., 2009a). Our study extends previously reported findings (Grabner et al., 2007; Fink et al., 2009a) since we included the aspect of time-course of idea generation. Regarding the earlier increase of alpha synchronization at right-hemispheric sites for higher original ideas, we assume that ideas of high quality require the same neural circuits and mechanisms than less original ideas, but probably in a more efficient manner, as indicated by an earlier and stronger increase in alpha synchronization of right-hemispheric sites compared to ideas of lower originality. For ideas of lower originality there is also an increase in alpha synchronization of right-hemispheric sites but only in the third time interval and the underlying idea generation process seems to be less advanced compared to ideas with higher idea originality. The resulting ideas might not be as elaborated as the more original ideas and are consequently rated as less original. This finding could thus be seen as additional support for the proposed interpretation of the EEG results.

As aforementioned, the present study is a re-analyses of a previous study of our laboratory (Fink et al., 2011). Therein, participants had to work on the AU task after being stimulated via affective and cognitive interventions and without any intervention. The analyses presented in this paper are based only on the trials of the control condition (i.e., without any intervention), and in this particular context we cannot completely rule out the possibility of spill-over effects (i.e., it may be possible that the interventions of the two other conditions may also have an unsystematic impact on the trials of the control condition). However, even though such kind of effects could possibly exist, it seems less likely that they were systematic since the trials were presented in a fully randomized order. Also, we cannot completely rule out the possibility that participants thought of more than one idea. However, behavioral analyses of idea fluency in this task showed that the average number of ideas within one minute is 4 (Benedek et al., 2013). As a consequence, a 10-s task should not elicit much more than 1 idea—particularly in view of the effect that participants were instructed to produce as original ideas as possible (i.e., instruction stressed quality, rather than fluency of ideas). We concede that people may still initially generate a dominant, typical response which is, however, rejected for the sake of finding creative ideas (Gilhooly et al., 2007). The brain activation related to such an initial response tendency was discussed to be associated with the earliest time epoch during idea generation. In addition, the analysis included the comparison of brain activity patterns associated with more vs. less original ideas which allowed for stronger and more powerful conclusions about the time-course of alpha activity during the process of creative idea generation.

To summarize, this study provides first insights into the time-course of creative ideation from the neuroscience perspective. In investigating task-related alpha power changes during creative idea generation we were able to describe some of the manifold cognitive processes implicated in the process of creative idea generation at the level of brain. As the findings of this suggest, the observed time course of alpha activity may reflect the progression of different stages in the process of idea generation: the idea generation process showed an initial bilateral alpha synchronization followed by a relative decrease in alpha power and an increasing hemispheric lateralization driven by a re-increase of alpha power at right frontal and posterior cortical sites. This is an entirely novel finding and it is proposed that the distinctive patterns of task-related alpha activity as a function of time reflect the sequence of well-known stages of the creative idea generation process: that is the initial retrieval of common and old ideas, followed by the actual generation of novel and more creative ideas by overcoming typical responses through processes of mental simulation and imagination (Gilhooly et al., 2007; Fink et al., 2009a; Benedek et al., 2011, 2014b; Fink and Benedek, 2012, 2013; Jauk et al., 2012).

## Conflict of interest statement

The authors declare that the research was conducted in the absence of any commercial or financial relationships that could be construed as a potential conflict of interest.
